# Glycoarray Technologies: Deciphering Interactions from Proteins to Live Cell Responses

**DOI:** 10.3390/microarrays5010003

**Published:** 2016-01-04

**Authors:** Tania M. Puvirajesinghe, Jeremy. E. Turnbull

**Affiliations:** 1 CRCM (Centre de Recherche en Cancérologie de Marseille), Cell Polarity, Cell Signalling and Cancer “Equipe labellisée Ligue Contre le Cancer”, Inserm, U1068, Marseille F-13009, France; taniap@liverpool.ac.uk; 2 Institut Paoli-Calmettes, Marseille F-13009, France; 3 Aix-Marseille Université, Marseille F-13284, France; 4 CNRS (Centre National de la Recherche Scientifique), UMR7258, Marseille F-13009, France; 5 Centre for Glycobiology, Department of Biochemistry, Institute of Integrative Biology, University of Liverpool, Liverpool L69 7ZB, UK

**Keywords:** glycomics, glycobioarrays, glycoconjugates, saccharide libraries, heparan sulfate

## Abstract

Microarray technologies inspired the development of carbohydrate arrays. Initially, carbohydrate array technology was hindered by the complex structures of glycans and their structural variability. The first designs of glycoarrays focused on the HTP (high throughput) study of protein–glycan binding events, and subsequently more in-depth kinetic analysis of carbohydrate–protein interactions. However, the applications have rapidly expanded and now achieve successful discrimination of selective interactions between carbohydrates and, not only proteins, but also viruses, bacteria and eukaryotic cells, and most recently even live cell responses to immobilized glycans. Combining array technology with other HTP technologies such as mass spectrometry is expected to allow even more accurate and sensitive analysis. This review provides a broad overview of established glycoarray technologies (with a special focus on glycosaminoglycan applications) and their emerging applications to the study of complex interactions between glycans and whole living cells.

## 1. Introduction

Carbohydrates are a major group of biomolecules, which can be subdivided into smaller families of molecules categorized by their structures. In natural states, carbohydrates are found conjugated to other biomolecules including proteins and lipids. Glycolipids are carbohydrates covalently attached to lipids and consist of important constituents including glycosphingolipids (GSLs), diacylglycolipids (DAGs) and lipopolysaccharides (LPs). Glycoproteins are formed when carbohydrates are conjugated to proteins via a serine or threonine residue (*O*-linked) or an asparagine residue (*N*-linked) depending on their distinct biosynthetic pathways. Proteoglycans represent a class of glycoconjugates richly dense in carbohydrates structures, which are generally long, unbranched molecules including glycosaminoglycans (GAGs), attached to a serine residue within the core protein via a xylose residue [1]. Free oligosaccharides also exhibit significant biological roles. One important example is milk glycans, whose expression pattern is highly regulated and controlled [2]. Bacterial polysaccharides and viral polysaccharides are another important form of biologically important glycan structures. Transient glycan variation has been documented in key physiological events including roles in pregnancy, lactation, infection, or acute phase response, and tissue and cell development [3]. The glycome describes the total collection of glycans synthesized by a cell, tissue, or organism under specified conditions of time, space, and environment [4]. Glycans vary in structure due to the high number of possible structural modifications, variable chain length of glycan chains and the fact that the biosynthetic process is not template driven and does not go to completion [5]. However, only a small proportion of each type of glycan structure has been found in mammalian systems. Therefore, the assumption is that nature does not require all possible glycan structures to function [6]. This has important consequences for the rationale design of glycan structures for glycan array platforms. Other important factors to consider with glycans are the flexibility of glycans due to anomericity; the ability of glycans to tightly cluster together to form three-dimensional internal structures (as documented from X-ray crystal structures) [7].

Introduction of array-based technology has significantly advanced the field of biology. In 1998, DNA microarrays heralded the introduction of an array platform, offering the simultaneous and high-throughput analysis of immobilized DNA molecules. Glycomic analyses seek to understand how a collection of glycans fulfil a range of particular biological functions. Therefore array-based technologies are particularly suited to the study of carbohydrate structures, providing the two main benefits, the need for low quantities of samples and achieving high-throughput parallel screening. Indeed this provided the rationale for the creation of the Consortium for Functional Glycomics (CFG) in 2001, which is now a widely used web-resource aimed at providing researchers with data, tools, resources, and information about community activities in the growing field of functional glycomics.

**Figure 1:**
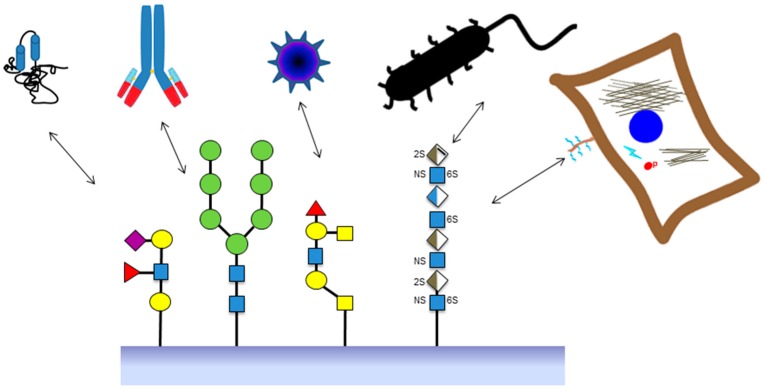
Schematic diagram of the various applications of glycan arrays relating to applications including screening protein and antibody interactions with various glycans, virus and bacteria interactions with glycoproteins; and interactions of mammalian cells with glycosaminoglycan structures which lead to live cell responses relating to phosphorylation of cell signaling cascades. Symbols for glycan structures use nomenclature from [7]. Yellow circles: galactose; yellow squares: *N*-acetylgalactosamine; blue squares: *N*-acetylglucosamine; green circles: mannose; blue/white diamonds: glucuronic acid; brown/white diamond: iduronic acid; sulphation shown by 2S, 6S and NS; extra lines on the diamond represent unsaturated bonds.

The use of microarrays for the study of carbohydrate interactions has lagged behind other biological molecules, with scientific literature appearing only in 2002 [8,9]. This is due to the wide structural variability of carbohydrate structures compared to other macromolecules and the difficulties in obtaining a large number of highly purified compounds. However, advances have been made in various areas including purification from natural sources, full chemical synthesis, immobilization and detection methods and automatization of sample handling. This has meant that carbohydrate arrays can now be used in screening molecular interactions as well as functional screening of whole organisms including eukaryotic cells, bacteria and viruses, and even living cell responses such as activation of cell signaling (Figure 1).

## 2. Sources of Glycan Structures

One major consideration for the construction of glycan arrays is the source of the glycoconjugate used, which in itself determines the aim of the experimental strategy. Sources of glycoconjugates can be broadly divided into three main subgroups: (1) Naturally occurring structures, which are extracted and purified from biological sources. Drawbacks of this source are that purification of glycans are hampered with factors such as multiple experimental steps associated with sample losses at every stage and reagents and conditions that can change the original glycan structures [10]. Methods adapted at using smaller sample sizes and providing more sensitive sample analysis circumvent these disadvantages [11]. For these reasons, carbohydrate arrays commonly use the latter two options of glycan libraries. (2) Libraries generated from natural glycans. Saccharide libraries can be generated by partial digestion of tissue-derived glycan chains and chromatographic fractionation of the resulting saccharide mixtures. Fractionation is initially based on the hydrodynamic volume, using size exclusion chromatography. Further fractionation, on the basis of charge or hydrophobicity can be used to separate the different glycan entities [12]. (3) Chemically-synthesized libraries. Traditionally, these libraries suffered from processes which were lengthy and needed highly specialized processes [13] and drawbacks included the fact that as the size of oligosaccharides increased, the yield of the coupling step decreased [14]. However, more rapid advances have been made in this type of technology with the use of enzymatic or chemoenzymatic *de novo* synthetic approaches. One example of the fundamental challenges of generating chemical libraries is the capacity to modify one specific hydroxyl group in the presence of many others. Strategies of glycan synthesis use steps to protect and mask chemical moieties in order to preferentially react the chemical groups of interest [15]. The second important consideration for glycan synthesis is the synthesis of the glycosidic bond. One strategy employed to generate glycosidic bonds is the use of recombinant glycosyltransferases [16]. The use of recombinant enzymes also means that the introduction of chemical groups such as sialic acid can be more easily accomplished as compared to chemical modification techniques [17]. The use of solid-support synthesis for glycans was inspired by the great advances previously made in peptide synthesis [18]. Translation of glycan synthesis to solid-phase platforms frequently employs the strategic placement of an amine-linker, which can in turn be covalently immobilized to glass surfaces [19]. Automation of the glycan chemical synthesis is now possible for several oligosaccharides on a solid-phase synthesizer [20], which is important in obtaining substances of high purity, a critical factor for the study of structure-activity relationships [21].

## 3. Choice of Solid Supports and Immobilization of Glycans onto Microarrays

Important factors to consider in the initial choice of solid supports for glycan arrays are twofold: whether derivatization of glycans is necessary and which type of chemistry is needed for immobilization of sugars onto the surface. However, this depends on the final experimental technique or the versatility of the resulting glycan array. Certain supports such as gold-based substrates provide advantages in that not only are they compatible for traditional fluorescence measurements using microarray scanners, but they can also be used as a platform for Matrix-assisted laser desorption ionization mass spectrometry (MALDI-MS), surface plasmon resonance (SPR), and quartz crystal microbalances [22]. The use of linkers, including polyethylene glycol also function as spacers and may have advantages in creating a separation from the matrix/plate surface [23].

There are different types of immobilization methods for glycans which exploit the large structural variety of glycoconjugates (see Table 1). One of the key components of a glycan array is the surface. Typical surfaces are microtiter plates, functionalized glass slides, nitrocellulose coated slides and gold slides [24,25,26,27]. The immobilization of the glycan structures onto a surface is the second consideration. Chemistries for the attachment of carbohydrates to surfaces can be broadly divided into four main categories, which are listed below: One criticism of glycan arrays is that the structure of oligosaccharides may affect the efficiency of their immobilization onto a surface, which may in turn affect the end-point signal measured [14]; in some platforms this has been largely addressed through production of tagged conjugates and their purification prior to immobilization. Examples include fluorescent labels such as 2-aminobenzamide (AB) or 2-aminobenzoid acid (AA) [28] and derivatives containing alkyl amines [29] or lipid tags [9].

### 3.1. Affinity Adsorption

One relatively straightforward method of immobilization of glycans onto a surface is adsorption. Glycans can be non-covalently and non-specifically immobilized onto nitrocellulose or oxidized black polystyrene surfaces [30,31]. Neoglyolipids can also be efficiently adsorbed onto nitrocellulaose slides [9]. Electrostatic interactions can be used to mediate immobilization between negatively charged glycans and surfaces coated with positively charged proteins such as poly-lysine [32].

### 3.2. Covalent Immobilization of Glycans

Modification of glycan structures can be time-consuming and costly; therefore, methods of immobilization that can use unmodified glycans are sometimes preferable. This type of immobilization can be achieved with photoactivatable supports which contain photolabile groups such as aryl(trifluoromethyl)diazirine [33]. Upon irradiation, photoproducts form intermediates of singlet carbene structure which can rapidly react with free glycan groups [34]. Immobilization of glycoconjugates and lectins has been achieved using this non-discriminatory type of immobilization [35]. The attachment of glycans via covalent bonds has been particularly useful for custom-based arrays such as heparin and heparan sulfate glycosaminoglycans, whereby the reducing-end aldehyde can be linked to amino and hydrazide surfaces groups on a surface [22,36,37,38,39].

**Table 1:** Different types of covalent attachment methods used for glycan arrays.

Type of Interaction	Type of Reaction	Details of Immobilization	Reference
Covalent	Condensation	Unmodified carbohydrates onto hydrazide surfaces.	[22,40,41,42]
Covalent	Michael Addition	Malemide-linked carbohydrates and thiol-coated glass slides.	[27,43]
Covalent	Epoxide opening	Covalently attach carbohydrates, glycoproteins and neoglycoconjugates to glass slides.	[44,45,46]
Covalent	Amide coupling	–	–
Covalent	Diels-Alder reaction	Covalent immobilization of glycans by cycloaddition.	[31]
Covalent	Carbene	Covalent immobilization of glycans by insertion.	[35]
Covalent	Radical coupling	Covalent immobilization of unmodified glycans by insertion.	[47]

### 3.3. Site-Specific Covalent Immobilization

The localization of glycan immobilization can be assured by using methods that result in covalent site-specific attachment of glycans. Immobilization methods based on the reaction between thiol and malemide groups are an example of covalent site-specific attachment of glycans. Studies have used malemide-conjugated sugars immobilized onto thiol-derivatized surfaces [27,43]. Conversely, thiol-linked sugars can be attached to malemide-coated surfaces [42,48]. Other types of covalent site-specific attachment include conjugation involving cyclopentadiene-linked sugars covalently attached to benzoquinone-coated surfaces [31]. In the field of GAG arrays, the ligation reaction between the aldehyde group and aminoxy or amino group has been crucial in the conjugation of heparin, heparin sulfate (HS) and chondroitin sulfate.

### 3.4. Non-Covalent Immobilization

One of the main disadvantages of covalent attachment to a monolayer is that high ligand concentrations are required which is somewhat quixotic when working with complex oligosaccharides [38]. Glycan structures with lipid tags allow the non-covalent immobilization onto MALDI plate by insertion into a self-assembled alkylthiolate monolayer. This simple and efficient procedure results in the orientated immobilization of glycans, and avoids the use of fluorescent tags [49].

## 4. Techniques for Detection of Protein Binding to Glycoarrays

The choice of surface is important as it determines the type of detection that can be used, as the detection method depends on the fundamental properties of the surface. The most common type of detection method of carbohydrate arrays relies on fluorescence detection and the use of fluorescently labeled proteins binding directly or indirectly to the glycan structures. The standard mode of detection in this case is a fluorescent microarray scanner. Fluorescence detection can be achieved using different methodologies: (1) using proteins labeled with fluorophores, (2) using fluorophores as a secondary reagent or (3) using fluorescent proteins that bind tags. The latter two sandwich assay options can be used to increase signal amplification as the number of fluorophores available for detection is increased [50]. Disadvantages in fluorescence detection are based on the drawbacks of fluorophores themselves, which include their sensitivity to light and the fragility to oxidative degradation. Carbohydrate arrays, which involve applications with cells, can utilize phase-contrast and fluorescence microscopy for detection steps. Acquisition of live-cell images is made possible by motorized microscope incubator stages which are designed to maintain physiological conditions for cell cultures during the image acquisition process. Other improvements in microscopy include increased automatization in autofocusing image stability and speed of image analysis [51]. Relative straightforward replacements such as replacing the light source with a programmable light emitting diode (LED) array of modules in standard microscope equipment can turn low-power microscopes into high-resolution imagers [52]. This can increase signal intensity, which can be an important factor in discriminating between different samples.

## 5. Use of Glycan Arrays for the High-Throughput Analysis of Glycan–Protein Interactions

As the cell membrane is a fluidic lipid bilayer environment, it is potentially important that the generation of glycan arrays mimic the lateral movement of glycans in their natural state at the cell membrane. Another important factor in protein–carbohydrate interactions is multivalency. Protein–carbohydrate interactions tend to be of low affinity but high specificity and so use multivalency to generate the affinity required for biologically relevant binding. In this way glycans can be organized to form clustered saccharide patches (CSPs) [53]. Applications that incorporate these important aspects include fluidic microarrays and CSP recognition in glycan microarrays.

Generic glycan arrays are available and allow the attachment of glycans from sources including mammals and microorganisms. One example of such a platform is described by Fukui *et al.* [9], which have exploited the attachment of oligosaccharides to lipid tails (neoglyolipids, NGLs) that can then be spotted on nitrocellulose membranes and consequently probed using proteins or peroxidase-conjugated lectins. Other formats have taken advantage of non-covalent yet strong interactions between biotin and streptavidin in order to conjugate biotinylated glycosides [54].

In comparison, custom-made arrays are also designed to answer specific questions. One example is the inclusion of the “designer microarray” with other combinatorial approaches to define the carbohydrate sequence of the Prostate Cancer-associated *Antigen* F77. The generation of designer NGLs probes from *O*-glycans was achieved by alkaline reductive release from a source of epithelial mucin, porcine stomach mucin (PSM). This method surprising found the F77 antigen to be expressed in blood group H on a 6-linked branch of a poly-*N*-acetyllactosamine backbone [55]. Another example of the application of the designer microarray approach pertains to glucanpolysaccharides, which are d-glucose polymers with differing linkages in linear or branched sequences and function as secreted virulence factors in bacteria. By using combinatorial approaches involving negative-ion electrospray tandem MS, information was obtained on linkage sequence and chain length requirement of glucan-recognition proteins such as Dendritic Cell-Specific Intercellular Adhesion molecule-3-Grabbing Non Integrin (DC-SIGN) [56]. Other examples of combinatorial arrays focus on the design of novel autoantibody targets formed from glycolipid and lipid complexes, formed from two or more individual species, can interact to create molecular shapes capable of being recognized by these autoantibodies [53,57,58]. A further example of custom-based arrays is based on the structure of heparan sulfates (see below).

## 6. Use of Glycan Arrays for Studying Heparin/Heparan Sulfate Interactions with Proteins

Heparan sulfate proteoglycans (HSPGs) contain protein core proteins that are covalently attached with HS chains. HS is a ubiquitous linear polysaccharide molecule, belonging to the GAG family of macromolecules. Attachment of HS to different core proteins results in HS having the ability to alter its location and topography as core proteins can occur at the cell surface and the extracellular matrix [59]. HSPGs are responsible for a multitude of different types of molecular interactions with different families of proteins and in different cellular contexts. HS is functionally important in many stages of the tumor process such as cellular transformation, tumor growth, invasion and metastasis. Factors which are important in modulating growth and metastasis are the charge density of HSPGs, level of expression of core proteins and the structure of the HS [60] Syndecan-1 is responsible for the maintenance of morphological differentiation and localization of epithelial cells. Additionally, a direct correlation between heparanases expression and the invasiveness of tumor cells has been shown [30]. Due to the interaction of HS with structural proteins such as laminin, HS can also provide physical barriers to tumor cells. In neurological contexts, HS also has important functions in Alzheimer’s disease [53,59] and roles in HIV attachment [61,62]. When located at the cell membrane, HSPGs interact with various growth factors, including members of fibroblast growth factors (FGFs), which are a large family of molecules (over 20 members), with similarities in sequence and functional properties [63]. The discovery that heparin binds acidic (FGF-1) and basic FGF (FGF-2) was made in 1989 by Thornton and coworkers who showed heparin potentiated the biological activity of crude preparations of acidic FGF [58]. The minimum sequence needed for HS binding to FGF2 contains a relatively common disaccharide structures in a pentasaccharide unit and consists of an *N*-sulfated glucosamine (GlcNS) and one Ido2S containing disaccharide [64]. Other examples show that more specificity is needed in the sequence of HS regulating its interactions. This is shown by the fact that a specific pentasaccharide sequence containing relatively rare modifications of 3*-O*-sulfate and the acetyl residue on an otherwise *N*-sulfated saccharide is responsible for heparin binding to increase the activity of antithrombin [65].

The minimal binding sequences are not always sufficient to increase biological activity. For FGF-2, twice the size of the minimum sequence is needed to produce a mitogenic response and a dodecasaccharide or longer sequence containing both IdoA (2-OSO_3_) and GlcNSO3(6-OSO_3_) residues is required [66,67]. Not all heparin binding proteins show this level of specificity. For example, hepatocyte growth factor/scatter factor (HGF/SF) is a 90 kDa paracrine factor synthesized by mesenchymal cells and involved in embryonic organ development and adult organ regeneration [68]. It has been shown that both HS and dermatan sulfate (DS) are both able to bind with a high affinity to a single common GAG-binding site and so have parallel abilities to modulate the HGF/SF induced functions [68]. Therefore it is unlikely that there is only one specific structure for binding and activity for any given protein and some structures can function with multiple proteins.

HS disaccharide units form long chain polysaccharide structures. There are at least 8 different common disaccharide structures that make up HS. For longer oligosaccharides, the number of variant structures increases exponentially [69]. However, the constraints of the biosynthetic process limit the number of possible structural variations. HS structures also have specific domain type structure that occurs in the full length HS polysaccharide chains, which typically varies between 50–200 disaccharide units long (that is equivalent to 25–100 kDa in size [14]. This manifests as regions of high sulfation, consisting of mainly IdoA residues sulfated at the 2 position (IdoA-2-S) and *N*-sulfated residues that are known as “S” or “NS” domain.

One of the main first applications of glycan arrays was to study how glycan structure would affect protein binding properties. This is being studied using arrays of heparin structures. Libraries containing heparin structures varying in their degree of sulfation as well as the length of their saccharide chains have been generated using heparin depolymerization [22] techniques in addition to chemical synthesis techniques [70]. The advantages of these assays are that, not only can quantitative data be generated on binding affinity but also the discovery of uncharacterized interactions can allow the investigation of novel therapeutic interventions. One example of the data generated using this type of approach is the measurement of relative binding affinities of different structures of heparin to FGF-1 and FGF-2 growth factors. This has shown that there is strong agreement between previously reported data [63]. Glycan arrays can then be used to calculate and determine the *Kd* values between proteins and immobilized glycans. The fluorescent intensities of bound proteins on glycan microarrays can be used to calculate the apparent dissociation constants [71]. Studies using the gold-standard approach for measuring *Kd* have shown that *Kd* values are similar to those determined using SPR experiments [72]. The measurement of proteins and glycans was originally used for the calculation of *IC*_50_ (half maximal inhibition) values for soluble inhibitors of proteins binding to glycans immobilized to surfaces. An example of the experimental setup includes a glycan array to which fluorescently labeled proteins are added, followed by soluble inhibitors. Following washing steps, the fluorescent intensities of the bound proteins are measured, which determines the *IC*_50_ values of the soluble inhibitor [73].

## 7. Glycoarrays for Measuring Glycan–Cell Interactions

Lectins are carbohydrate-binding proteins and macromolecules that are highly specific for sugar moieties. Lectins are present in plants, microbes and animals [4]. Lectins use specific carbohydrate-binding domains (CRD) to bind to carbohydrates. Glycan–lectin interactions mediate various processes notably in innate immunity and pathogenesis of viruses [74]. Carbohydrate microarrays have been used to identify and compare the binding preferences of different lectins, including C-type lectins. DC-SIGN, which is a receptor that plays a dual role in interaction of dendritic cells with pathogen surfaces as well as with T cells [75,76], was one of the first receptors tested against glycan arrays. Glycan arrays showed that DC-SIGN was able to bind high-mannose structures, in addition to fucose-terminated sugars including Lewis A and Lewis B structures [77]. Cell adhesion has been quantitatively assessed using glycan arrays. This has been shown using glycan arrays with lectin structures on hepatocytes. It has also been shown using glass slides with covalently attached monosaccharides and oligosaccharides of non-reducing terminal *N*-acetylglucosamine (GlcNAc) residues, galactose (Gal) and *N*-acetylgalactosamine residues. Primary chicken hepatocytes express a well-defined C-type lectin that binds to non-reducing terminal *N*-acetylglucosamine residues, and was labeled with a fluorescent dye. A specific chamber was used to remove non-adherent cells (GlycoChip^®^ Centrifugation Chamber, Agilent Technology, Santa Clara, California, USA) and adherent cells were measured using fluorescence detection. Chicken hepatocytes bound selectively to lectins derivatized with GlcNAc structures rather than spots of lectin with Gal or no modifications [78].

## 8. Glycoarrays for Measuring Virus and Bacteria–Glycan Interactions

Glycan arrays have been very important in the understanding of surface interaction of various other microorganisms including interactions with viruses. The interaction of envelope glycoproteins with protein binding partners has been the basis of the development of the design of vaccines, which has been extensively exploited to interfere with the interaction involving the entry of the HIV virus. Glyco-protein microarrays have been used to analyze the glycan interactions dictating the interaction between the two HIV-1 (human immunodeficiency virus) glycoproteins, gp-120 and gp-41. The structures decorating the viral-surface envelope glycoproteins of HIV include high-mannose oligosaccharides including triantennary mannoside-1 [79]. Synthesized glycan structures contain a thiol-terminated ethylene glycol linker which allow attachment of the sugars onto a malemide-functionalized glass slide using the stable covalent bond [80]. This has made possible the identification of HIV vaccine candidate antigens [81].

Carbohydrate–protein interactions also dictate interactions between human cells and other pathogens including various bacteria such as *Helicobacter pylori* and *Escherichia coli*. Initial interactions determine adherence, which is an important factor of pathogenicity in microorganisms and separate human microbiota from pathogenic bacteria [82]. Carbohydrate microarray studies on glycan–bacteria interactions can also be exploited for screening for attachment inhibitors that can be used as novel antibiotics, as well as serving as detection platforms which can detect as well as purify certain bacterial species which are capable of differential expression of glycan-binding proteins [71]. Immobilization techniques employ ethanolamine linkers at the reducing end of synthesized glycans in combination with CodeLink slides. These slides are coated with a hydrophilic polymer containing *N*-hydroxylsuccinimide (NHS) ester reactive group and so enable the covalent attachment of sugars [83]. After hybridization of bacteria, cells can be stained with cell-permeant fluorescent nuclei staining dyes such as SYTO 62. These carbohydrate arrays have shown the ability to successfully identify species of bacteria which specifically express mannose-receptors and bind mannose sugars immobilized to surfaces [84]. Therefore the large scale screening of bacteria can be used to isolate and identify species of bacteria which are potentially pathogenic, in order to delineate a subspecies of bacteria which can be further analyzed [85].

Carbohydrate arrays can also be used to characterize aminoglycoside antibiotics. Aminoglycosides contain aminocyclitol rings with one or more amino sugars. Aminoglycosides bind to various sites including the A site of 16S rRNA of bacteria as well as other RNA species [86,87,88,89] and lead to the alteration of translation at diverse steps including initiation, elongation and termination and therefore inhibit bacterial protein synthesis. One of the major drawbacks of aminoglycosides is the increasing resistance in bacteria [90]. Aminoglycoside arrays have been useful in screening different RNA structures from bacteria including *Candida albicans*, which show the ability to bind aminoglycoside structures. In addition, differential binding can be detected by varying amounts of fluorescence.

## 9. Glycoarrays for Reporting Live Cell Responses Including Cellular Signaling Pathways

**Figure 2:**
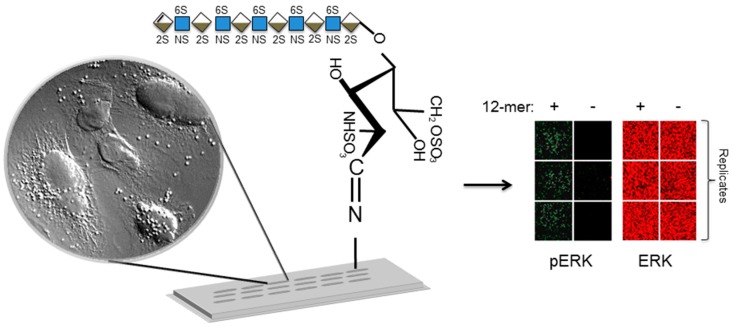
Schematic diagram of a glycobioarray platform for screening live cell fibroblast growth factor signaling responses to immobilized heparin saccharides. See Puvirajesinghe *et al.* [39] for details. Saccharides immobilized onto an aminosilane glass surface via a Schiff’s base linkage with their reducing ends, is shown. Cells (shown using 40× magnification) can be overlaid onto the slide surface and cultured for a specified period, followed by fixation and immunostaining to detected specific epitopes for phosphorylation (green fluorescence for phosphorylated ERK and red fluorescence for total ERK) events using a microarray slide scanner. Symbols for glycan structures use nomenclature from [7]. Blue squares: *N*-acetylglucosamine; blue/white diamonds: glucuronic acid; brown/white diamond: iduronic acid; sulphation shown by 2S, 6S and NS; extra lines on the diamond represent unsaturated bonds.

The interactions of specific glycan structures with proteins may produce various functional consequences such as activation or inhibition of cellular processes. This means that functional assays are very important in addition to the protein interaction measurements; recent work has shown that glycoarrays can also be used to assess living cell responses to immobilized glycans. For example in the HS structure-function paradigm, many studies have analyzed the effects of HS structure on long-term cellular outcomes, such as cell proliferation, using standard cell biology techniques. Recently however, the direct investigation of the effect of different HS structures on cellular signaling in a glycoarray format has been described for the first time [39]. Previous studies have shown that chain length of HS influence the interaction with FGF-2 and affect ERK1/2 signaling events and eventual outcomes of FGF-2 signaling such as cell proliferation. ERK1/2 can be activated or switched on without the presence of HSPG, however, this effect is transient [91,92]. This is typical of the behavior of ERK1/2 which has been described to act as a continuously variable switch that controls transcription [93] in diverse cellular programs including embryogenesis, proliferation, differentiation and apoptosis [94]. The activation of ERK1/2 is critical in G_0_-G_1_-S phase progression due to regulation of cyclin D1. Mitogens such as FGF2 and particular HS saccharides structures in chlorate- treated Swiss 3T3 cells evoke a biphasic increase in ERK1/2 activity involved a large initial increase in activity followed by a degree of activity sustained at a lower level. This pathway could be studied in-depth by the development of a high-throughput (HTP) assay which allowed study of the functional effect of many HS structures simultaneously. 96-well plate assays have been used effectively in the past [95]. Puvirajesinghe *et al.* [39] have described a cell-responsive glycoarray format—termed “glycobioarrays”—which allow analysis of cell signaling responses of cells overlaid on spotted glycans on glass slides (Figure 2). Glycobioarrays provide an innovative platform to analyze the consequences of stimulation using different structures of glycans in terms of the activation of different signaling cascades. The data obtained in proof-of-concept studies on HS activation of FGF signaling corresponded to that reported in literature, where dp10 and dp12 heparin and HS oligosaccharides are sufficiently large to induce a functional response in cells [96,97,98]. This platform has potential for wider application with glycoarrays for measurement of cell responses requiring extracellular interactions of cell surface proteins with their regulatory glycan targets.

More recently, the study of molecular interactions dictating cellular processes such as cellular proliferation has now been made possible using 3D platforms and the use of 3D block printing. This has resulted in the development of high-throughput miniaturized 3D-chip platforms that can examine the importance of specific structures in HS and CS (chondroitin sulfate) in ternary structures of growth factor and growth factor receptor signaling complexes [99]. 3D block printers have been used to print immortalized bone marrow (BaF3) cells that have no HSPGs on the cellular surface and express a single FGFR [63,100], which makes them an ideal cellular model for the use with exogenous addition of FGF growth factor and HS structures. In order to translate 96-well cell proliferation assays to an array-based platform, the following experimental setup was used. Acid-washed glass slides were first modified with polystyrene co-malic anhydride and dried. Onto the slide, spots of BaCl_2_ and polylysine were arrayed onto the surface and then BaF3 cells in a viscous solution of alginate were then spotted onto the polylysine spots. Soluble growth factors and exogenous structures of HS and chondroitin sulfate (CS) were added to the media. The mechanism of FGF-FGFR-GAG signal transduction and how it relates to cellular activity can be examined by assessing cell proliferation using reagents which distinguish live cells from dead cells and assess intracellular esterase activity and plasma membrane integrity. Calcein AM and ethidium homodimer (EthD-1) dyes were used for this application [101,102,103]. This type of assay means that numerous combinations of FGF growth factors and GAG structures can be performed in parallel and in replicates [99]. A second important factor is that glycan microarray platforms require the use of lower quantities of reagent [99].

## 10. Interrogation of Glycoarrays Using Mass Spectrometry

Coupling mass spectrometry (MS) with glycan arrays provides a promising application for the discovery of new glycan-binding ligands and binding proteins. To date, the main achievements in this field have been made with respect to lectin microarrays. Applications include the systematic identification of carbohydrate-binding proteins in proteomes [104]. One such example of a lectin glycan array is based on the covalent immobilization of lectins onto a molded silicone polymer, polydimethylsiloxane (PDMS) by the use of oxidation of PDMS, silanization with aminopropyltrimethoxysilane and cross-linking with glutaraldehyde [41]. The high degree of flexibility of PDMS enables the substrate to be easily attached to a MALDI plate for MALDI-MS measurement, following incubation of lectin arrays with patient sera [41]. Mass spectrometry is also a powerful method for defining the saccharides arrayed in “designer” glycome arrays produced from biological sources [56].

## 11. Conclusions and Future Perspectives

Key advances in glycan arrays over the past two decades have been in screening carbohydrate-binding proteins in proteomes, calculating protein binding affinities and automatization of solid-support synthesis for glycans. Integration and examination of this wealth of information is beginning to become more standardized with the use of public data repositories within consortia such as the CFG. Aspects of immobilization, choice of support and detection have now been studied for different types of glycans depending on the type of application. Technological developments mean that increased sensitivity as well as combinatorial approaches exploiting mass spectrometry techniques for glycan array interrogation will permit new methodological advances to continue. Further exploitation of glycobioarrays may also provide higher throughput functional level screening of glycan activities. Collectively, these will improve the quality and amount of data, plus improve quantitation of data. Ultimately, this will yield a wealth of insights into the functional diversity and functional specificity of glycans and will underpin new routes to exploit this knowledge in biomedical applications.
